# Dimensions of the psychological emptiness and its relation with suicide history among college students

**DOI:** 10.3389/fpsyg.2025.1626912

**Published:** 2025-07-03

**Authors:** Xing Hang, Wenqi Lü, Yan Wu, Xudong Kang, Ye Zhu

**Affiliations:** ^1^Business School, Chengdu University, Chengdu, China; ^2^Sleep Medicine Center, West China Hospital, Sichuan University, Chengdu, China; ^3^Department of Postgraduate Student, West China School of Medicine, Sichuan University, Chengdu, China; ^4^College of Marxism, Sichuan University, Chengdu, China; ^5^Mental Health Education Center, Chengdu University, Chengdu, China; ^6^School of Marxism, University of Electronic Science and Technology of China, Chengdu, China

**Keywords:** psychological, psychiatry, suicide, psychopatalogy, behavior

## Abstract

**Background:**

The psychological emptiness (PE) presents a state of emotional emptiness and lack of meaning, and is increasingly relevant in modern society. However, few studies have tried to assess its dimension and suicide history. This study aims to establish the factorial structure of PE and compare its predictive power against a standard depression inventory.

**Methods:**

A total of 45,335 university students participated in the study. The 20 items involving lack of purpose, depression, and meaninglessness in life were used to evaluate the PE, alongside the Beck Depression Inventory (BDI). Exploratory factor analysis (EFA) was employed to identify the structure of the 20 items, while confirmatory factor analysis (CFA) was employed to confirm the construct validity of the model. Logistic regression analysis was conducted to assess the predictive relationship between the identified factors and suicide history.

**Results:**

EFA identified a three-factor structure: Depression and Self-Harm/Suicidal Tendencies (DST), Life Meaning and Purpose (LMP), and Study Motivation (SM). The three factors accounted for 23.1, 12.6, and 11.9% of the total variance, respectively. CFA confirmed the construct validity of the model, which showed acceptable fit indices and high internal consistency (Cronbach’s *α* > 0.80). Logistic regression analysis revealed that DST was the strongest predictor of suicide risk (AUC = 0.84), outperforming traditional depression scales (BDI, AUC = 0.58).

**Conclusion:**

The present study provides a comprehensive framework for understanding PE. The PE may include three psychological dimensions, while DST is a strong predictor of suicide history.

## Introduction

Psychological emptiness (PE) refers to a state of inner emptiness, meaninglessness, and a lack of purpose in life, similar to the concept of existential vacuum, a term first introduced by Viktor Frankl (Valdés-Stauber et al., 2023). While commonly encountered in clinical practice, PE remains poorly understood due to the absence of a consensus definition, lack of standardized and validated assessment instruments, and limited empirical investigation into its phenomenology and longitudinal course (D'Agostino, 2020). Recently, some studies have tried to create a validated definition of PEA. They identified a two-factor structure encompassing ‘nothingness’ and ‘detachment’ (Herron and Sani, 2022; Herron et al., 2024). However, the exploration of PE’s underlying dimensions is still in the early stages.

Suicide remains one of the leading causes of death among university students, posing significant challenges to mental health and well-being (Gselamu and Ha, 2020; Bornheimer et al., 2022). Among the various psychological factors contributing to suicide risk, existential issues including the experience of PE, should also be considered (Herron et al., 2024). PE has been associated with mental health problems, including depression, anxiety, and suicidal tendencies (Valdés-Stauber et al., 2023). It is not merely an abstract philosophical concept but a tangible psychological state with far-reaching implications for mental health (Bornheimer et al., 2022). Notably, many individuals who die by suicide do not have a diagnosed psychiatric condition, suggesting the presence of other significant risk factors (such as PE). PE may manifest as emotional distress, alienation, self-doubt, and even self-harm or suicidal ideation, reflecting risks beyond traditional mental health diagnoses like depression or anxiety (Bendassolli, 2017). Factors contributing to this crisis include the pressure to conform to societal expectations (Imataka and Shiraishi, 2024), the pervasive influence of digital media, and the erosion of traditional sources of meaning, such as community, spirituality, and personal relationships (Giumetti and Kowalski, 2022; Serrano and Dolci, 2021). Thus, focusing on the multidimensional nature of PE, including potential factors such as depression, self-harm tendencies, life meaning and purpose, and achievement motivation, may provide a more comprehensive understanding of this phenomenon.

In this study, we hypothesize that PE is a multidimensional construct, with each dimension resulting in a different risk of suicide. To test this hypothesis, we will use exploratory factor analysis (EFA) to examine the structure of the PE, followed by confirmatory factor analysis (CFA) to validate the identified factors. Subsequently, logistic regression analysis will be employed to assess the predictive relationship between the identified factors and suicide risk.

## Methods

### Participants

This study was conducted among current college students who agreed to complete the questionnaire. Participants who did not fully complete the questionnaire were excluded. Ethical approval (No. 2018015) was granted by the Chengdu University Psychology Research Ethics Committee and was conducted in accordance with the Declaration of Helsinki. Participants were invited to complete the questionnaire via a secure online platform. On accessing the survey link, each student was first presented with an information page detailing the study’s objectives, procedures, potential risks and benefits, data confidentiality, and their right to withdraw at any time without penalty. To indicate their voluntary agreement, participants clicked an ‘I have read and agree to participate’ button; only those who provided this online consent were able to proceed to the questionnaire.

### Assessment of depressive symptoms

The Beck Depression Inventory (BDI) was used to evaluate depressive symptoms in participants. The BDI is a widely utilized self-report questionnaire designed to measure the severity of depressive symptoms over the past 2 weeks. It consists of 21 items, each scored on a scale from 0 to 3, with total scores ranging from 0 to 63. Depressive symptoms were categorized as minimal (0–13), mild (14–19), moderate (20–28), or severe (29 or above). This study employed the standardized and validated Chinese version of the BDI, which demonstrates strong reliability and validity (Shek, 1990).

### Assessment of PE and suicide history

The self-developed 20 items involving lack of purpose, depression, and meaninglessness in life were used to evaluate the PE. The 20 items were rated on a 5-point (1 = strongly disagree, 5 = strongly agree). Higher scores indicate greater levels of the items. The full items are presented in Supplementary Table S1 (Table 1). The suicide history was measured by a single dichotomous question: ‘I have had experiences of suicide’ (yes/no).’

**Table 1:** Demographics of included participants.

Items	N (%) or mean (SD)
Total (Female)	45,335 (24,432, 53.89%)
Age	22.10 (2.66)
Education	
Undergraduate	40,690 (89.75%)
Postgraduate	4,645 (10.25%)
UNESCO classification	
Medical and Health Sciences	4,247 (9.37%)
Engineering and Technology	16,553 (36.51%)
Humanities	6,842 (15.09%)
Social Sciences	16,757 (36.96%)
Others	936 (2.06%)
BDI	6.51 (6.31)
5–7 mild	8,031 (17.71%)
8–15 moderate	11,680 (25.76%)
≥16 several	4,343 (9.58%)
Suicide history	1,434 (3.16%)

### Exploratory factor analysis

The EFA was conducted using the maximum likelihood (ML) estimation method with Oblimin rotation to identify the underlying factor structure of the PE (Lin and Yao, 2022). The optimal number of factors was determined by evaluating the scree plot, the Kaiser-Guttman criterion (eigenvalues > 1), and the cumulative explained variance. The dataset included responses from a training sample (a random subset of 50% of the data) to explore the 20-items factor of the PE. Items with factor loadings approximately ≥0.40 were considered significant contributors to their respective factors. Each item was assigned to the factor on which it had the highest standardized loading.

### Confirmatory factor analysis

The CFA was performed to validate the factor structure derived from the EFA using the verification dataset (rest of 50% of the data) with the lavaan package in R (McNeish and Wolf, 2023). Model fit was evaluated using the Comparative Fit Index (CFI), Tucker–Lewis Index (TLI), Root Mean Square Error of Approximation (RMSEA), and Standardized Root Mean Square Residual (SRMR). CFI and TLI values ≥ 0.90 were interpreted as good fit, values between 0.85 and 0.90 as an acceptable fit. RMSEA values ≤ 0.05 were considered a good fit, values between 0.05 and 0.09 as an acceptable fit. SRMR values ≤ 0.05 were considered a good fit, values between 0.05 and 0.08 as an acceptable fit. To further probe the dimensionality of the scale, two additional CFA models were specified. Including the Second-Order Factor Model (SOFM) and Bifactor Model (BM). In SOFM, the three factors identified by EFA each load onto a single higher-order factor (G). This tests whether the intercorrelations among F1, F2, and F3 can be accounted for by one overarching construct. The BM specifies a general factor (G) on which all 20 items load, and three orthogonal group factors (F1, F2, F3) that load only on their respective item subsets. Covariances between G and each group factor, as well as among group factors, are constrained to zero to ensure a pure bifactor structure.

### Internal consistency reliability

Internal consistency reliability for each factor was assessed using Cronbach’s *α* (Stewart et al., 2001). A Cronbach’s α > 0.70 was considered acceptable. The analysis was conducted on the verification dataset. For each factor, Cronbach’s α was calculated, and the effect of removing individual items on α was examined to evaluate their contributions.

### Statistical analysis

Continuous measurements were presented as mean (standard deviation, SD) and categorical variables as count scale, and the model performance was evaluated using AUC (Area Under Curve), which quantifies the model’s differentiating ability. The pROC package was used to compute and plot the ROC curves and AUC values for each model. A higher AUC value indicates better differentiating ability. For each factor, we performed logistic regression to examine its relationship with suicide risk. The AUC of the ROC curve was calculated for each model to compare their differentiating performance (Lü et al., 2023). ROC curves were plotted for each factor to visually assess their effectiveness in differentiating participants with and without a suicide history.

## Result

### Demographics

**Figure 1:**
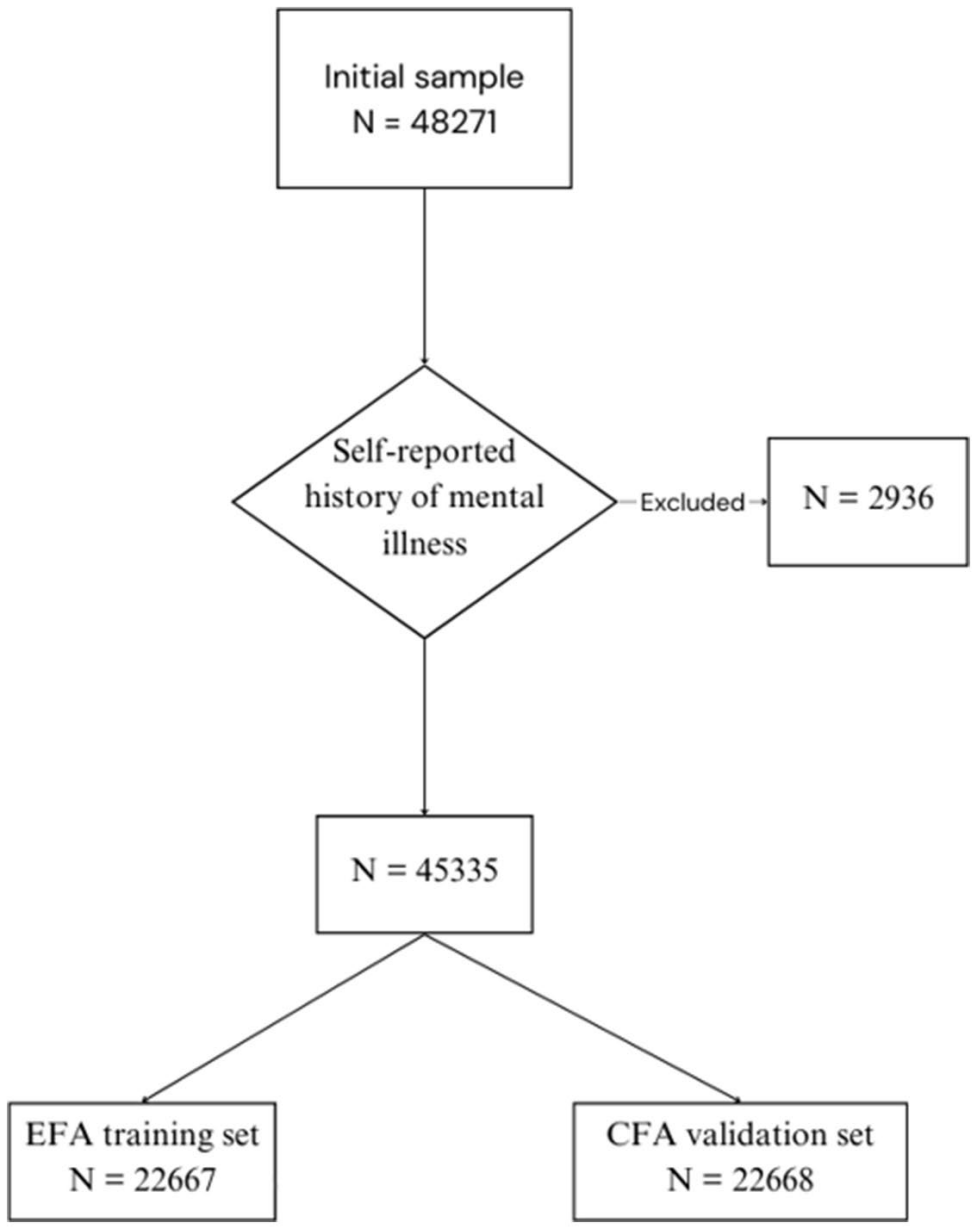
Flow diagram.

**Figure 2:**
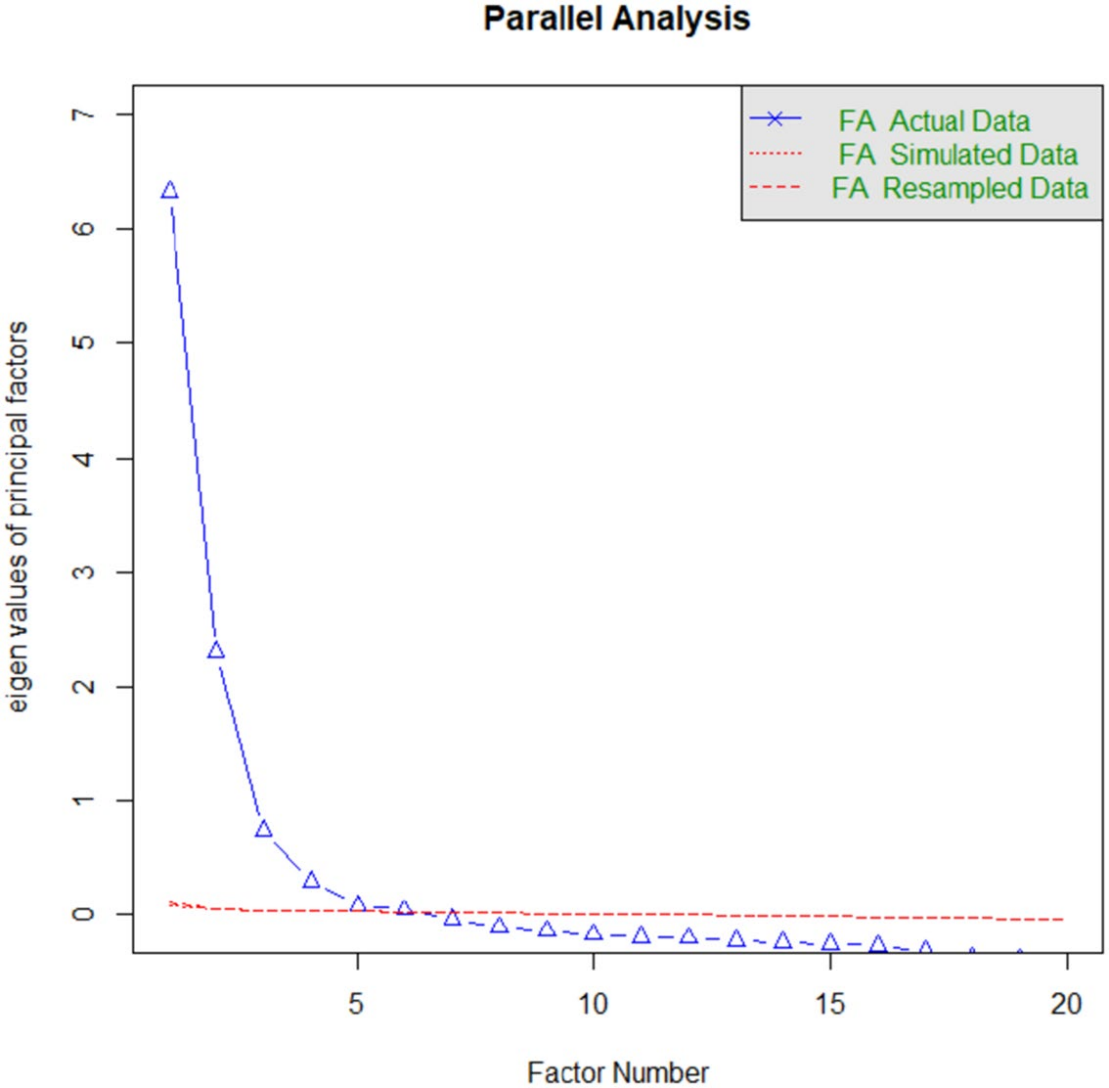
Scree plot of EFA.

A total of 48,271 college students completed the questionnaire, while 2,936 participants were excluded due to self-reported history of mental illness (Figure 1). Consequently, a total of 45,335 participants were enrolled in this study. The sample included 24,432 (53.89%) females, with a mean age of 22.10 years (SD = 2.66). Regarding education levels, the majority of students were undergraduate (40,690, 89.75%), while 4,645 participants (10.25%) were postgraduate. The mean BDI score was 6.51 (SD = 6.31). Based on BDI thresholds, 17.71% (*n* = 8,031) of participants exhibited mild depressive symptoms (scores 5–7), 25.76% (*n* = 11,680) displayed moderate symptoms (scores 8–15), and 9.58% (*n* = 4,343) presented with severe depressive symptoms (scores ≥16). Suicide history was reported by 3.16% individuals. Using the UNESCO classification system, participants were distributed across various academic disciplines: Medical and Health Sciences (*n* = 4,247), Engineering and Technology (*n* = 16,553), Humanities (*n* = 6,842), Social Sciences (*n* = 16,757), and other fields (*n* = 936; Figure 2).

### Exploratory factor analysis

**Figure 3:**
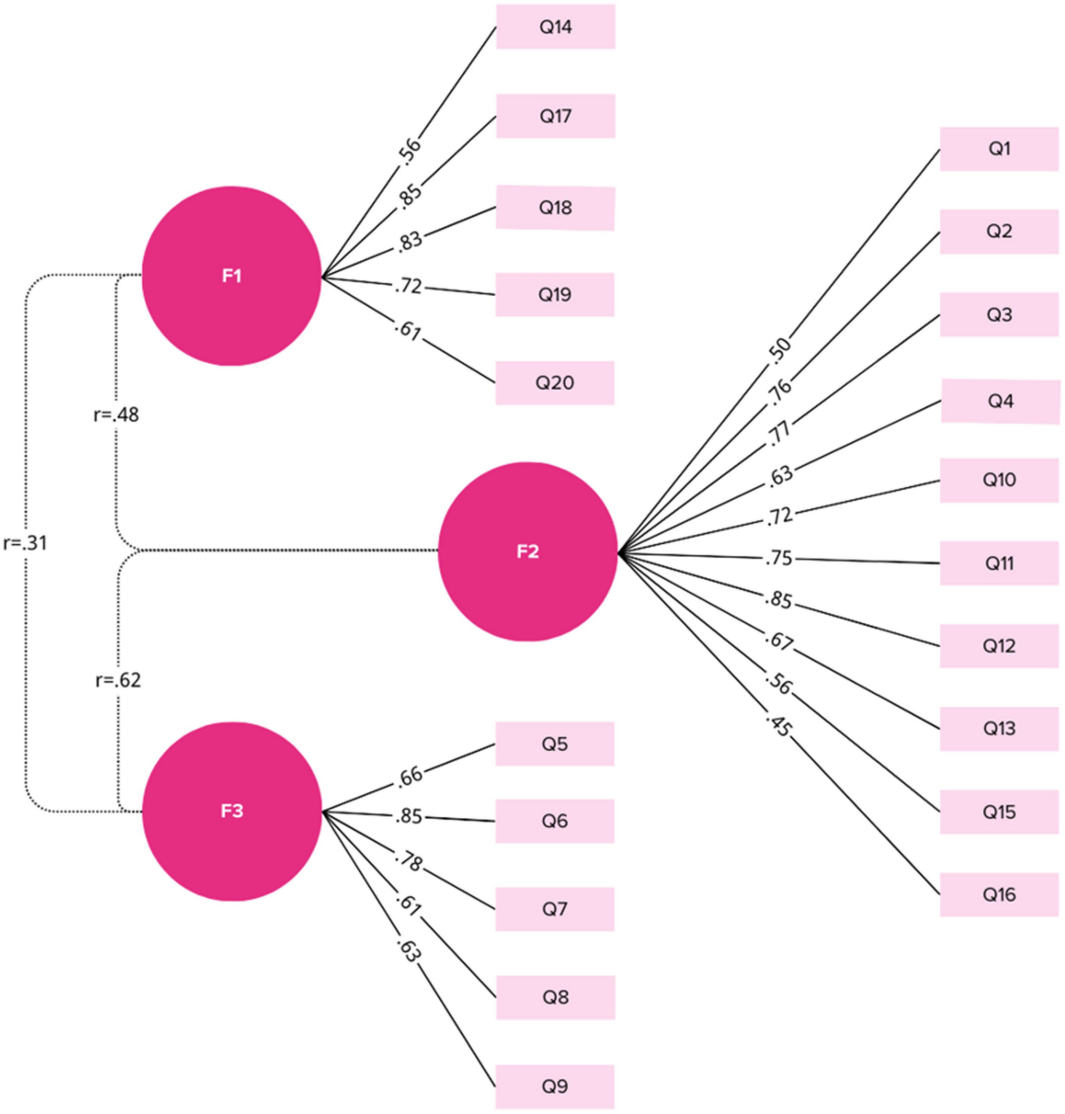
Factor structure with loadings and correlations. The circle represents the factor and the rectangle represents the entry; the numbers on the path are standardized factor loads; the dashed line shows the correlation between the factors.

A sample size of 22,667 was used for the EFA. The Keiser–Meyer–Olkin value was 0.92, and Bartlett’s test of sphericity chi-square was 220718.60 (df = 190), *p* < 0.001, which indicates the data were appropriate for EFA. The scree plot indicated a clear inflection point at the third factor, suggesting a 3-factor solution. Based on the pattern matrix of the EFA results and a thorough review of the item content, the three factors were designated as follows: Factor 1 was named “Depression and Self-Harm/Suicidal Tendencies” (DST), encompassing items related to emotional distress, self-harm behaviors, and suicidal thoughts. Factor 2 was named “Life Meaning and Purpose” (LMP), representing items that reflect an individual’s sense of purpose, value, and hope in life. Factor 3 was named “Study motivation” (SM), covering items that assess an individual’s attitudes toward the study. The three factors accounted for 47.7% of the total variance, with the F1 (DST) contributing 23.1%, the F2 (LMP) 12.6%, and the F3 (SM) 11.9% (Table 2; Figure 3).

**Table 2:** Items used to evaluate psychological emptiness and their factor loadings.

Items	Factor1 loading	Factor 2 loading	Factor 3 loading	Primary factor
Q1. I am often willing to help others		0.525		F2
Q2. I have a clear understanding of the meaning of my life		0.809		F2
Q3. My life has clear goals		0.825		F2
Q4. Learning is meaningful to me	−0.159	0.656	0.224	F2
Q5. Learning makes me upset		0.102	0.660	F3
Q6. I hate studying and try to avoid it			0.904	F3
Q7. Studying is meaningless	0.121		0.747	F3
Q8. I tend to study hard but hate it at the same time		0.131	0.530	F3
Q9. I have been feeling low for more than a month	0.365		0.388	F3
Q10. I feel hopeful about my future.		0.669		F2
Q11. I feel that I am useful and indispensable		0.723		F2
Q12. My life is very meaningful	0.113	0.791		F2
Q13. I still find interest in the things I usually enjoy doing		0.619		F2
Q14. I feel very lonely, and no one truly understands me	0.377	0.179	0.189	F1
Q15. I have good relationships with the people around me		0.556		F2
Q16. I have people whom I admire and respect		0.446		F2
Q17. I sometimes think about ending my life	0.881			F1
Q18. I do not know why I am living	0.685	0.130	0.128	F1
Q19. I have tried to harm myself.	0.752			F1
Q20. When life has no value, suicide is understandable	0.606			F1

### Confirmatory factor analysis and construct validity

**Figure 4:**
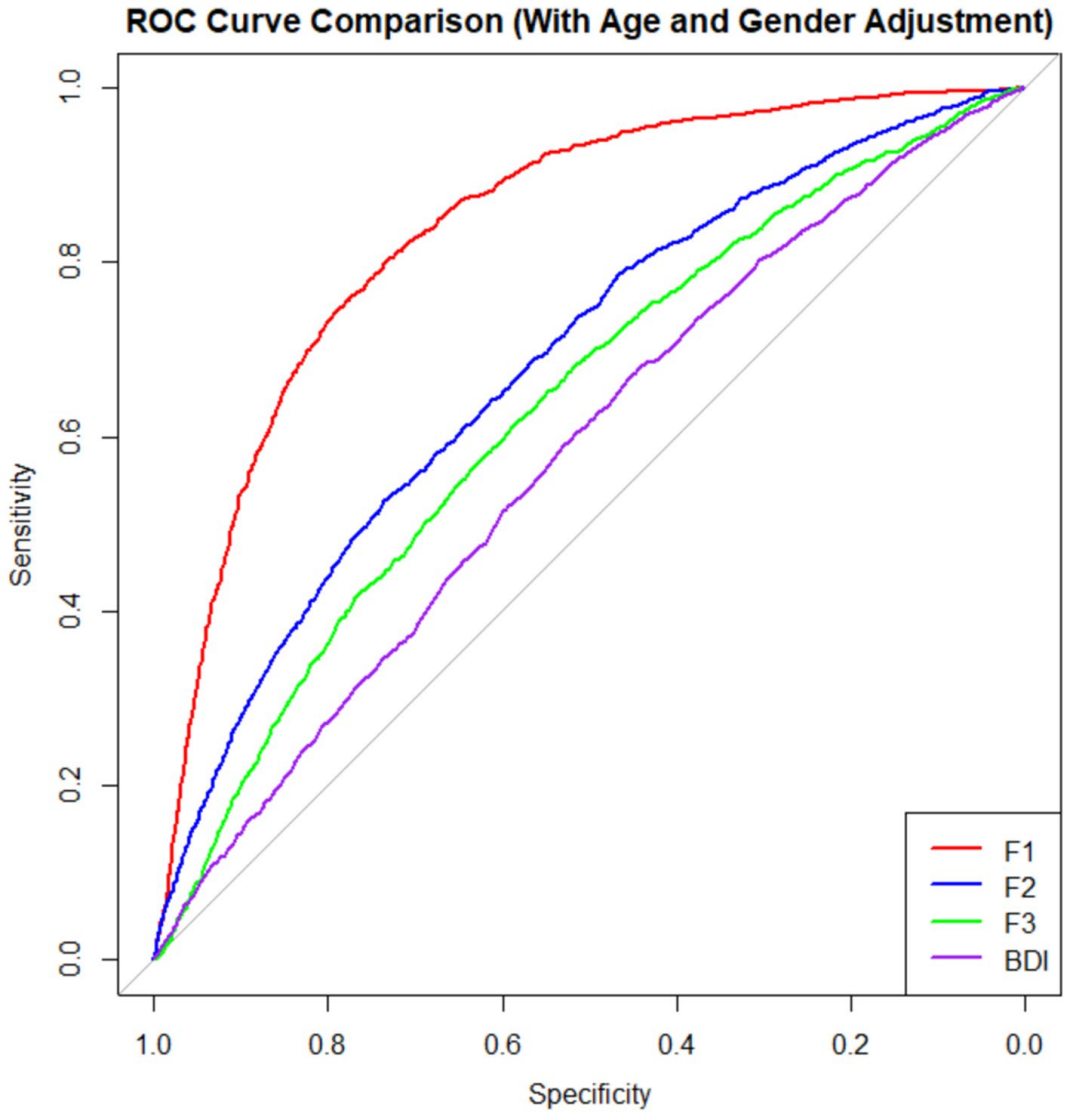
ROC curve analysis of identified factors and BDI. AUC for F1: 0.8390525; AUC for F2: 0.68237; AUC for F3: 0.632943; AUC for BDI: 0.5784164.

The three-factor model demonstrated acceptable fit: with CFI: 0.872; TLI: 0.854; RMSEA: 0.086; and SRMR: 0.065. The standardized factor loadings demonstrated that all items loaded significantly on their respective factors, with standardized loadings ranging from 0.52 to 0.90, meeting the threshold of 0.40. The Cronbach’s alpha coefficients for the F1, F2, and F3 were 0.89, 0.83, and 0.82, respectively. For all factors, removing individual items did not result in a significant increase in Cronbach’s *α*, indicating all items contributed meaningfully to their respective factors (Table 3; Supplementary Table S2). The SOFM demonstrated comparable fit to the original first-order solution (CFI = 0.872; TLI = 0.854; RMSEA = 0.086; SRMR = 0.062; Figure 4), indicating that a single higher-order factor did not substantially improve model fit over the three-factor structure. Additionally, a BM was estimated, which provided a better fit (CFI = 0.919; TLI = 0.898; RMSEA = 0.072; SRMR = 0.079; Supplementary Table S3). Schmid–Leiman decomposition yielded an explained common variance (ECV) of 0.42 for the general factor and a hierarchical omega (ωₕ) of 0.54, while group-specific omegas (ωₛ) were 0.69 for F1(DST), 0.19 for F2 (LMP), and 0.47 for F3(SM). These results confirm the presence of a strong overarching dimension alongside three meaningful subdimensions, supporting reporting both the total score and subscale scores in future applications.

**Table 3:** Internal consistency reliability of identified factors.

Factor	Cronbach’s α	Average correlation (r)
F1	0.83	0.50
F2	0.89	0.45
F3	0.82	0.49

### Relationship with suicide history

The present study performed logistic regression for each factor (F1, F2, F3, BDI) with age and gender as covariates. The AUC values were 0.84, 0.68, 0.63, and 0.58 for F1, F2, F3, and BDI, respectively. This indicated that F1 (DST) exhibited the strongest ability to distinguish between individuals with and without a suicide history, significantly outperforming F2 (LMP), F3 (SM), and BDI in differentiating suicide history.

## Discussion

This study provides novel insights into the multidimensional nature of PE and its relation with suicide history among college students with no self-reported history of mental illness. By applying EFA and CFA, we identified a three-factor structure for PE: DST, LMP, and SM. The findings suggest that these dimensions of PE are distinct, with DST exhibiting the strongest association with suicide history, outperforming BDI.

A growing body of evidence suggests that PE is not a singular, monolithic construct, but rather a multidimensional experience that encompasses various psychological aspects. A previous study developed a PE scale that targeted people living with personality disorders, which identified two factors named nothingness and detachment (Herron et al., 2024). Our study may provide a more comprehensive understanding of PE among college students. The LMP factor emphasizes the critical role of a sense of purpose and meaning in buffering against psychological distress. The DST factor underscores the emotional burden and self-destructive tendencies associated with PE. Finally, the factor of SM reflects the inner conflict and dissatisfaction individuals may experience in their pursuit of personal or career goals. These dimensions collectively provide a more comprehensive understanding of PE as both an emotional and cognitive phenomenon.

The PE demonstrated excellent psychometric properties, with satisfactory internal consistency (Cronbach’s *α* for all factors > 0.80) and construct validity, as evidenced by the results of confirmatory factor analysis. The model fit indices indicate an acceptable fit to the data, while a bifactor model provided a superior fit, indicating both a general dimension and meaningful subdomains. Future research should explore scoring and validating both the overall scale and its subscales. Generally, these findings establish the identified factors of PE as a reliable and valid instrument for assessing PE. We also highlight that students who have reported suicide history should draw great attention from educational institutions. Previous studies demonstrated that school connectedness is protective of suicidality, and more protective of suicidal ideation than suicide attempts (Welty et al., 2024). Moreover, institutions may provide individualized prevention according to the PE dimension. For example, individuals scoring high on DST may benefit from suicide prevention strategies, while those with low scores on LMP may respond well to meaning-centered therapies.

The results of the logistic regression analysis demonstrated that DST had the strongest predictive ability for suicide risk among the three factors and the BDI. These findings indicate that DST is significantly associated with lower suicide risk and demonstrates a stronger association with suicide-related outcomes than traditional measures such as the BDI. The high AUC value for DST indicates that emotional distress and self-harm behaviors are strongly associated with suicide risk (Rotenstein et al., 2016; Ribeiro et al., 2018). This finding aligns with the extensive literature that identifies hopelessness, self-harm, and suicidal ideation as primary predictors of suicidal behavior (Ribeiro et al., 2018). The superior predictive ability of DST suggests that future research may investigate whether incorporating focused assessments and interventions for negative affect and self-harm tendencies can enhance suicide-prevention outcomes.

Despite its lower AUC (0.68) compared to DST, LMP remains a meaningful predictor of suicide risk. The results suggest that a diminished sense of life meaning and purpose may indirectly contribute to suicidal tendencies by exacerbating feelings of hopelessness and emotional distress (Marco et al., 2020). A previous network study also found that life meaning is an important central node for suicide (Guo, 2023). Interventions designed to enhance life meaning, such as meaning-centered therapies, may serve as protective factors against suicide. SM demonstrated similar predictive power for suicide.

Our findings revealed that the BDI demonstrated a relatively weak ability to predict suicide history (AUC = 0.58), not only compared to the DST, but also LMP and SM. One possible reason for the relatively low AUC of the BDI in predicting suicide history is that the BDI contains few items directly related to self-harm and suicidal behaviors (Shek, 1990). As a result, the BDI may not fully capture the emotional and behavioral indicators that are critical in assessing suicide risk. Additionally, the very name of the BDI may increase participants’ vigilance and lead to response bias. A previous study conducted an experimental study in which they compared a standard, overtly labeled BDI with a covertly described version of the same inventory (Hunt et al., 2003). Participants in the covert condition reported significantly more core depressive symptoms than those receiving the overt label, suggesting that explicit naming can lead to underreporting due to stigma and self-presentation motives. The term “depression” may carry a stigma, causing some participants to underreport their depressive symptoms or suicide history due to fear of judgment or social consequences (Devendorf et al., 2020). This could result in a lower predictive power of the BDI compared to other scales that more directly assess self-destructive tendencies and existential distress (Sharma, 2024). Future work should incorporate instruments such as the social desirability scale and explicitly reinforce confidentiality and anonymity to detect and control for this bias.

The present study has several strengths. To our knowledge, this is the first study to explore the dimension of PE among college students with no self-reported history of mental illness. Additionally, the study included a large sample size and provided a broad and diverse representation of the student population, which may benefit from the strong reliability of the results. Moreover, we also tried to link the dimension factors of PE and suicide history, providing an additional perspective on suicide risk factors. However, there are also several limitations to consider. First, the cross-sectional design limits the ability to predict future suicide risk, and the retrospective nature of suicide history reporting may introduce recall bias. Additionally, the study primarily focused on the relationship between the dimensions of PE and suicide history. Future studies should explore how these dimensions might relate to other psychological health outcomes such as academic performance, life satisfaction, career development, anxiety, or resilience. Moreover, the present study did not include measures of province of origin, urban–rural residency, or ethnicity. Future studies should collect and report these demographic variables to examine potential regional and cultural differences. Finally, we acknowledge that the BDI was selected as our primary comparator because of its widespread use and well-established validity for assessing depressive symptom severity. However, the BDI is not designed specifically to capture suicidal ideation, and future studies should include suicide-focused instruments (e.g., the Beck Scale for Suicide Ideation) to more directly benchmark DST’s predictive value. In addition, unmeasured confounders such as variations in social support and trauma history may have differentially attenuated the BDI’s predictive power and warrant careful control in subsequent prospective investigations.

## Data Availability

The original contributions presented in the study are included in the article/Supplementary material, further inquiries can be directed to the corresponding author.

## References

[ref1] Bendassolli P. F. (2017). Emptiness and work: a meaning-making perspective. Integr. Psychol. Behav. Sci. 51, 598–617. doi: 10.1007/s12124-017-9382-x, PMID: 28150138

[ref2] Bornheimer L. A. Czyz E. Koo H. J. Li Verdugo J. Eisenberg D. Zheng K.. (2022). Suicide risk profiles and barriers to professional help-seeking among college students with elevated risk for suicide. J. Psychiatr. Res. 152, 305–312. doi: 10.1016/j.jpsychires.2022.06.028, PMID: 35772258 PMC10653046

[ref3] D'Agostino A. (2020). The feeling of emptiness: a review of a complex subjective experience. Harv. Rev. Psychiatry 28, 287–295. doi: 10.1097/HRP.0000000000000269, PMID: 32773487

[ref4] Devendorf A. Bender A. Rottenberg J. (2020). Depression presentations, stigma, and mental health literacy: a critical review and YouTube content analysis. Clin. Psychol. Rev. 78:101843. doi: 10.1016/j.cpr.2020.101843, PMID: 32304914

[ref5] Giumetti G. W. Kowalski R. M. (2022). Cyberbullying via social media and well-being. Curr. Opin. Psychol. 45:101314. doi: 10.1016/j.copsyc.2022.101314, PMID: 35313180

[ref6] Gselamu L. Ha K. (2020). Attitudes towards suicide and risk factors for suicide attempts among university students in South Korea. J. Affect. Disord. 272, 166–169. doi: 10.1016/j.jad.2020.03.135, PMID: 32379610

[ref7] Guo Z. (2023). The relationships between suicidal ideation, meaning in life, and affect: a network analysis. Int. J. Ment. Heal. Addict. 7, 1–20. doi: 10.1007/s11469-023-01019-9 PMC9904259 36776916

[ref8] Herron S. J. Sani F. (2022). Understanding the typical presentation of emptiness: a study of lived-experience. J. Ment. Health 31, 188–195. doi: 10.1080/09638237.2021.1922645, PMID: 34008477

[ref9] Herron S. J. Saunders R. Sani F. Feigenbaum J. (2024). The psychological emptiness scale: a psychometric evaluation. BJPsych Open 10:e42. doi: 10.1192/bjo.2023.649, PMID: 38299317 PMC10897692

[ref10] Hunt M. Auriemma J. Cashaw A. C. (2003). Self-report bias and underreporting of depression on the BDI-II. J. Pers. Assess. 80, 26–30. doi: 10.1207/S15327752JPA8001_10, PMID: 12584064

[ref11] Imataka G. Shiraishi H. (2024). Youth suicide in Japan: exploring the role of subcultures, internet addiction, and societal pressures. Diseases 13:2. doi: 10.3390/diseases13010002, PMID: 39851466 PMC11764159

[ref12] Lin L. C. Yao G. (2022). Validation of the factor structure of the WHOQOL-BREF using meta-analysis of exploration factor analysis and social network analysis. Psychol. Assess. 34, 660–670. doi: 10.1037/pas0001122, PMID: 35298219

[ref13] Lü W. Zhang M. Yu W. Kuang W. Chen L. Zhang W.. (2023). Differentiating Alzheimer's disease from mild cognitive impairment: a quick screening tool based on machine learning. BMJ Open 13:e073011. doi: 10.1136/bmjopen-2023-073011, PMID: 38070931 PMC10729043

[ref14] Marco J. H. Cañabate M. Llorca G. Pérez S. (2020). Meaning in life moderates hopelessness, suicide ideation, and borderline psychopathology in participants with eating disorders: a longitudinal study. Clin. Psychol. Psychother. 27, 146–158. doi: 10.1002/cpp.2414, PMID: 31765024

[ref15] McNeish D. Wolf M. G. (2023). Dynamic fit index cutoffs for confirmatory factor analysis models. Psychol. Methods 28, 61–88. doi: 10.1037/met0000425, PMID: 34694832

[ref16] Ribeiro J. D. Huang X. Fox K. R. Franklin J. C. (2018). Depression and hopelessness as risk factors for suicide ideation, attempts and death: meta-analysis of longitudinal studies. Br. J. Psychiatry 212, 279–286. doi: 10.1192/bjp.2018.27, PMID: 29587888

[ref17] Rotenstein L. S. Ramos M. A. Torre M. Segal J. B. Peluso M. J. Guille C.. (2016). Prevalence of depression, depressive symptoms, and suicidal ideation among medical students: a systematic review and Meta-analysis. JAMA 316, 2214–2236. doi: 10.1001/jama.2016.17324, PMID: 27923088 PMC5613659

[ref18] Serrano C. C. Dolci G. F. (2021). Suicide prevention and suicidal behavior. Gac. Med. Mex. 157, 547–552. doi: 10.24875/GMM.M21000611, PMID: 35104269

[ref19] Sharma V. (2024). Prevention of self-harm and suicide in young people up to the age of 25 in education settings. Cochrane Database Syst. Rev. 2024:Cd013844. doi: 10.1002/14651858.CD013844.pub2, PMID: 39704320 PMC11660227

[ref20] Shek D. T. (1990). Reliability and factorial structure of the Chinese version of the Beck depression inventory. J. Clin. Psychol. 46, 35–43. 2303562 10.1002/1097-4679(199001)46:1<35::aid-jclp2270460106>3.0.co;2-w

[ref21] Stewart W. F. Lipton R. B. Dowson A. J. Sawyer J. (2001). Development and testing of the migraine disability assessment (MIDAS) questionnaire to assess headache-related disability. Neurology 56, S20–S28. doi: 10.1212/WNL.56.suppl_1.S20, PMID: 11294956

[ref22] Valdés-Stauber J. Böttinger J. Kramer S. Kämmerle H. (2023). Differences in life attitudes between general population and hospitalized psychosomatic patients: a comparative cross-sectional study. Psychol. Health Med. 28, 1729–1740. doi: 10.1080/13548506.2022.2120624, PMID: 36052986

[ref23] Welty C. W. Bingham L. Morales M. Gerald L. B. Ellingson K. D. Haynes P. L. (2024). School connectedness and suicide among high school youth: a systematic review. J. Sch. Health 94, 469–480. doi: 10.1111/josh.13445, PMID: 38383772

