# Supplementary material for: Dimensions of the psychological emptiness and its relation with suicide history among college students
Source: Front Psychol. 2025 Jul 3;16:1626912. doi: 10.3389/fpsyg.2025.1626912 (PMC12269772; doi:10.3389/fpsyg.2025.1626912)
Supplement: Supplementary file 1 [file Data_Sheet_1.docx]

S Table 1 The 20-items used to evaluate PE

| Items |
| --- |
| Q1. I am often willing to help others |
| Q2. I have a clear understanding of the meaning of my life |
| Q3. My life has clear goals |
| Q4. Learning is meaningful to me |
| Q5. Learning makes me upset |
| Q6. I hate studying and try to avoid it |
| Q7. Studying is meaningless |
| Q8. I tend to study hard but hate it at the same time |
| Q9. I have been feeling low for more than a month |
| Q10. I feel hopeful about my future. |
| Q11. I feel that I am useful and indispensable |
| Q12. My life is very meaningful |
| Q13. I still find interest in the things I usually enjoy doing |
| Q14. I feel very lonely, and no one truly understands me |
| Q15. I have good relationships with the people around me |
| Q16. I have people whom I admire and respect |
| Q17. I sometimes think about ending my life |
| Q18. I don’t know why I am living |
| Q19. I have tried to harm myself. |
| Q20. When life has no value, suicide is understandable |

S Table 2 Cronbach’s α for each factor and impact of item removal

| Factor | Item | Cronbach’s α if the item removed | Original Cronbach’s α |
| --- | --- | --- | --- |
| Factor 1 | VQ14 | 0.83 | 0.83 |
|  | VQ17 | 0.76 | 0.83 |
|  | VQ18 | 0.77 | 0.83 |
|  | VQ19 | 0.78 | 0.83 |
|  | VQ20 | 0.81 | 0.83 |
| Factor 2 | VQ1 | 0.89 | 0.89 |
|  | VQ2 | 0.87 | 0.89 |
|  | VQ3 | 0.87 | 0.89 |
|  | VQ4 | 0.88 | 0.89 |
|  | VQ10 | 0.88 | 0.89 |
|  | VQ11 | 0.88 | 0.89 |
|  | VQ12 | 0.87 | 0.89 |
|  | VQ13 | 0.88 | 0.89 |
|  | VQ15 | 0.89 | 0.89 |
|  | VQ16 | 0.89 | 0.89 |
| Factor 3 | VQ5 | 0.79 | 0.82 |
|  | VQ6 | 0.75 | 0.82 |
|  | VQ7 | 0.78 | 0.82 |
|  | VQ8 | 0.80 | 0.82 |
|  | VQ9 | 0.80 | 0.82 |

S Table 3 Comparison of fit indices for first-order, second-order, and bifactor models

| Model | CFI | TLI | RMSEA | SRMR |
| --- | --- | --- | --- | --- |
| First-order model | 0.872 | 0.854 | 0.086 | 0.065 |
| Second-order model | 0.872 | 0.854 | 0.086 | 0.062 |
| Bifactor model | 0.919 | 0.898 | 0.072 | 0.079 |

**Abbreviations:** CFI: Comparative Fit Index; TLI: Tucker–Lewis Index; RMSEA: Root Mean Square Error of Approximation; SRMR: Standardized Root Mean Square Residual
